# Hydrogen Inhalation Protects against Ototoxicity Induced by Intravenous Cisplatin in the Guinea Pig

**DOI:** 10.3389/fncel.2017.00280

**Published:** 2017-09-13

**Authors:** Anette E. Fransson, Marta Kisiel, Kristian Pirttilä, Curt Pettersson, Pernilla Videhult Pierre, Göran F. E. Laurell

**Affiliations:** ^1^Department of Surgical Science, Uppsala University Uppsala, Sweden; ^2^Division of Analytical Pharmaceutical Chemistry, Department of Medical Chemistry, Uppsala University Uppsala, Sweden; ^3^Division of Audiology, Department of Clinical Science, Intervention and Technology, Karolinska Institutet Stockholm, Sweden

**Keywords:** ABR, inner hair cells, outer hair cells, synaptophysin, organic cation transporter 2, copper transporter 1, perilymph metabolomics, *in vivo*

## Abstract

**Introduction:** Permanent hearing loss and tinnitus as side-effects from treatment with the anticancer drug cisplatin is a clinical problem. Ototoxicity may be reduced by co-administration of an otoprotective agent, but the results in humans have so far been modest.

**Aim:** The present preclinical *in vivo* study aimed to explore the protective efficacy of hydrogen (H_2_) inhalation on ototoxicity induced by intravenous cisplatin.

**Materials and Methods:** Albino guinea pigs were divided into four groups. The Cispt (*n* = 11) and Cispt+H_2_ (*n* = 11) groups were given intravenous cisplatin (8 mg/kg b.w., injection rate 0.2 ml/min). Immediately after, the Cispt+H_2_ group also received gaseous H_2_ (2% in air, 60 min). The H_2_ group (*n* = 5) received only H_2_ and the Control group (*n* = 7) received neither cisplatin nor H_2_. Ototoxicity was assessed by measuring frequency specific ABR thresholds before and 96 h after treatment, loss of inner (IHCs) and outer (OHCs) hair cells, and by performing densitometry-based immunohistochemistry analysis of cochlear synaptophysin, organic transporter 2 (OCT2), and copper transporter 1 (CTR1) at 12 and 7 mm from the round window. By utilizing metabolomics analysis of perilymph the change of metabolites in the perilymph was assessed.

**Results:** Cisplatin induced electrophysiological threshold shifts, hair cell loss, and reduced synaptophysin immunoreactivity in the synapse area around the IHCs and OHCs. H_2_ inhalation mitigated all these effects. Cisplatin also reduced the OCT2 intensity in the inner and outer pillar cells and in the stria vascularis as well as the CTR1 intensity in the synapse area around the IHCs, the Deiters' cells, and the stria vascularis. H_2_ prevented the majority of these effects.

**Conclusion:** H_2_ inhalation can reduce cisplatin-induced ototoxicity on functional, cellular, and subcellular levels. It is proposed that synaptopathy may serve as a marker for cisplatin ototoxicity. The effect of H_2_ on the antineoplastic activity of cisplatin needs to be further explored.

## Introduction

Hearing disabilities can be the result of exogenous factors such as exposure to noise and treatment with drugs that have ototoxic side effects. Oncologic treatment using cisplatin-based therapy, is a common cause of drug-induced hearing loss, especially when high doses are given. Cisplatin is among the first generation of platinum-based anticancer agents and is widely used to treat different tumors in both children and adults. Cisplatin can enter the cochlea (Laurell et al., 1995; Hellberg et al., 2013), likely via passive membrane diffusion and active uptake to vulnerable cells. The details of this transport are still not fully elucidated, but organic cation transporter 2 (OCT2) (Ciarimboli et al., 2010) and copper transporter 1 (CTR1) (More et al., 2010) have been implicated in cisplatin uptake to the inner ear.

Inside the cochlea, cisplatin can damage the organ of Corti, spiral ganglion, stria vascularis, and spiral ligament (van Ruijven et al., 2005; Rybak, 2007). At the cellular level, cisplatin-induced ototoxicity typically manifests as loss of outer hair cells (OHCs), mainly in the basal turn of the cochlea, resulting in high-frequency hearing loss (Rybak, 2007). Effects on subcellular compartments are less understood. Recent data suggest that cochlear synaptopathy is a key to acquired hearing loss (Liberman and Kujawa, 2017); however, its role in cisplatin-induced ototoxicity is unknown.

Preclinical experiments suggest that underlying mechanisms of cisplatin-induced ototoxicity include the formation of platinum-DNA adducts, inflammation, and oxidative stress (Rybak, 2007). A number of experimental studies have shown that it is possible to reduce cisplatin ototoxicity by co-administrating an otoprotective drug (Rybak, 2007). However, no otoprotective therapy has clearly attenuated cisplatin ototoxicity in clinical practice.

Pharmacological treatment of the inner ear can involve either systemic or local drug administration, and both have advantages and disadvantages. Systemic administration is a well-established method that can be non-invasive (e.g., oral) or invasive (e.g., intravenous). One challenge is that the cochlea possesses at least two barrier systems, the blood-perilymph and intrastrial fluid-blood barriers (Cohen-Salmon et al., 2007; Shi, 2016), that prevent drugs in systemic circulation from accessing the cochlear compartments. Another challenge is that systemic distribution of a drug may lead to unwanted effects in other systems. Unwanted effects of antioxidant treatment to prevent cisplatin-induced ototoxicity include inhibition of the antineoplastic efficacy of cisplatin (Freyer et al., 2017), stimulation of tumor growth (Sayin et al., 2014), and metastasis (Le Gal et al., 2015). These effects are unacceptable in humans with cancer but difficult to study in experimental models. The literature offers limited information in this area, although there are several experimental studies describing otoprotective (Rybak, 2007) and nephroprotective (dos Santos et al., 2012) effects with concomitant use of drugs with antioxidative effects. An alternative to systemic administration is local drug delivery, which makes it possible to achieve a high drug concentration in the target organ, thereby reducing the risk of unwanted effects in other parts of the body. Some pharmacological agents that have been applied systemically have also been tested for intratympanic administration. Local treatment of the inner ear can only be performed with invasive techniques, and its clinical use is currently limited to steroid treatment for sudden sensorineural hearing loss after failure of systemic steroid treatment (Li et al., 2015) and destructive treatment of vertigo with gentamicin in Ménière's disease (Pullens and van Benthem, 2011).

Multiple candidate substances, mostly antioxidants, have been tested to prevent cisplatin-induced ototoxicity, but little is known about the importance of different physicochemical properties for otoprotective efficacy. One interesting candidate is molecular hydrogen (H_2_) as it is expected to easily penetrate the different cochlear barriers and reach the organ of Corti. H_2_ can be given as a gas or an aqueous solution, and it has been shown to have antioxidant (Fukuda et al., 2007; Ohsawa et al., 2007; Xie et al., 2010) and anti-inflammatory (Gharib et al., 2001; Xie et al., 2010) properties. Gaseous H_2_ is not explosive at a concentration <4% in air or pure oxygen (Huang et al., 2010). Inhaled H_2_ reduced organ damage in different animal models of ischemia (Fukuda et al., 2007; Ohsawa et al., 2007; Huang et al., 2011; Hugyecz et al., 2011). Importantly, cisplatin-induced nephrotoxicity was attenuated by aqueous H_2_ in rats (Kitamura et al., 2010; Matsushita et al., 2011) and by aqueous and gaseous H_2_ in mice (Nakashima-Kamimura et al., 2009), likely by reducing oxidative stress (Nakashima-Kamimura et al., 2009). With regard to effects related to hearing, aqueous H_2_ reduced antimycin A-induced production of reactive oxygen species (ROS) and increased the survival of inner hair cells (IHCs) and OHCs in a cochlear explant study (Kikkawa et al., 2009). In a gerbil model of auditory neuropathy, gaseous H_2_ reduced ouabain-induced hearing threshold shifts and spiral ganglia damage (Qu et al., 2012a). In guinea pig models of noise-induced hearing loss, aqueous H_2_ was shown to reduce hearing threshold shifts (Lin et al., 2011; Zhou et al., 2012; Chen et al., 2014) and hair cell loss (Zhou et al., 2012; Chen et al., 2014), likely by alleviating oxidative stress (Chen et al., 2014, 2017) and/or inflammation (Chen et al., 2017). Gaseous H_2_ also reduced noise-induced hearing threshold shifts, OHC loss, and oxidative stress in a guinea pig study (Kurioka et al., 2014). An important advantage of H_2_ is that it is considered safe for human use (Huang et al., 2010).

Based on existing findings, we presumed that H_2_ therapy exerts different protective effects at subcellular levels in the inner ear when given in conjunction with systemic cisplatin. The present study aimed to study the otoprotective effects of gaseous H_2_ in guinea pigs treated with cisplatin by evaluating auditory brainstem response (ABR) threshold shifts, hair cell loss, and immunohistochemical findings in subcellular structures damaged by cisplatin.

## Materials and methods

### Study design

Guinea pigs (*n* = 34) were randomly assigned to four treatment groups: Cispt (*n* = 11), Cispt+H_2_ (*n* = 11), H_2_ (*n* = 5), or Control (*n* = 7). The Cispt and Cispt+H_2_ groups received a single intravenous (i.v.) injection of cisplatin. In the Cispt+H_2_ group, this injection was immediately followed by 60-min administration of gaseous H_2_. The H_2_ group only received 60-min administration of H_2_, while the Control group received neither cisplatin nor H_2_. Auditory function was assessed with frequency-specific ABR measured prior to and approximately 96 h after cisplatin administration. After the final ABR measurement, the Cispt and Cispt+H_2_ groups were subjected to perilymph sampling, after which they were euthanized with a high dose of pentobarbital. The H_2_ and Control groups were euthanized immediately after the final ABR measurement. Finally, the animals' cochleae were collected for histological analyses. Two of the animals in the Control group were used for ABR recordings, and five animals were processed for immunohistochemistry.

### Animals

Guinea pigs (Duncan-Hartley, Lidköpings Kaninfarm, Lidköping, Sweden) of both sexes, 5–8 weeks old and weighing 250–504 g were used. The animals were kept in an enriched environment and housed in small groups with lights on between 7 a.m. and 7 p.m. at a temperature of 21°C and a humidity of 60%. They were given free access to water and standard chow and were allowed to acclimatize for at least 10 days before the experiment started. All procedures were performed under anesthesia and aseptic conditions. The guinea pigs had normal tympanic membranes and hearing as determined by otoscopic examination and ABR assessment. During the experimental procedures, the animals were placed on a homeothermic pad. Body weight was measured daily. The Cispt and Cispt+H_2_ groups were hydrated with sterile saline (5 ml, 37°C) subcutaneously (s.c.) each day after cisplatin exposure. All animal procedures were performed in accordance with the ethical guidelines of Uppsala University and consistent with national regulations for animal care and use (ethical permit C 106/13; Uppsala's ethical committee on animal experiments).

### Anesthesia

All experiments were performed on anesthetized animals. General anesthesia was achieved with ketamine (40 mg/kg b.w.; Ketalar, 50 mg/ml; Pfizer AB, Sweden) and xylazine (10 mg/kg b.w.; Rompun, 20 mg/ml; Bayer Health Care AG, Denmark) intramuscularly. The depth of anesthesia was determined by measurement of the pedal reflex, and additional doses of ketamine (25 mg/kg b.w.) were given if needed. Bupivacaine (Marcain, s.c., 2.5 mg/ml, AstraZeneca, Sweden) was used as local anesthesia, and buprenorphine, (0.06 mg, Temgesic, 0.3 mg/ml, s.c.; Schering-Plough, NJ, USA) was used as a post-treatment analgesic in animals subjected to cisplatin administration.

### Cisplatin administration

Cisplatin (8 mg/kg b.w.; Platinol 1 mg/ml; Bristol-Myers Squibb AB, Sweden) was administered at an injection rate of 0.2 ml/min through a catheter (PE50, ID = 0.58 mm, OD = 0.965 mm, Intramedic Clay Adams Brand; Becton Dickinson and Company, NJ, USA) inserted into the right jugular vein toward the heart. Immediately after, 1 ml sterile saline was administered through the same catheter. The catheter was then removed, the jugular vein was ligated, and the skin was sutured (Ethilon II polyamide 4-0).

Regulations from the Swedish Work Environment Act (AFS 2005:5) were followed during cisplatin handling and destruction.

### H_2_ administration

H_2_ was administered over 60 min using a gas mixture of H_2_ (2 mol%), oxygen (O_2_; 21 mol%), and nitrogen (N_2_; 77 mol%; AGA Gas AB, Sweden). The gas was delivered through a facial mask. The flow rate was set at 0.5 l/min using a single-stage pressure regulator (C 200/1 A B 3 BAR DIN 1, Linde AG, Linde Gases Division, Germany).

### ABR

Frequency-specific ABR at 3.15, 6.30, 12.5, 20.0, and 30.0 kHz was recorded to monitor auditory function. The animal was anesthetized as previously described and placed in a soundproof box. The stimulus signal was generated through a signal analyzer (Tucker-Davis Technologies, FL, USA) controlled by a personal computer and presented through an electrostatic speaker (EC1; Tucker-Davis Technologies, FL, USA). The speaker was connected to a 10-cm tube positioned in the guinea pig's ear canal. Neural responses were collected using three subdermal electrodes: one placed at the vertex (active), one placed on the mastoid (reference), and a ground electrode placed at the lower back. The ABR threshold was determined as the lowest stimulus intensity that produced a reproducible response for ABR wave II, which was visualized at the same latency after an average of 1,000 recordings.

#### Metabolomics analysis of scala tympani perilymph

About 1 h after the last ABR measurement, animals in the Cispt and Cispt+H_2_ groups underwent perilymph sampling from the basal turn of the cochlea as previously described (Hellberg et al., 2013). Perilymph (1 μL) was collected from both ears, diluted 1:19 with water, and stored at −80°C until further handling. Some samples were excluded due to contamination or insufficient volume. Samples were thawed in the refrigerator overnight and subjected to protein precipitation by adding cold acetonitrile (4:1 acetonitrile to perilymph, kept on ice) and centrifuged (4°C, 21,000 RCF, 15 min). The supernatant was stored at −80°C pending analysis and analyzed without further treatment. A quality control (QC) sample was prepared as a mixture of aliquots from all samples. Analysis was performed on a UHPLC-Q-ToF-instrument (Waters, Milford, MA, USA). Data were acquired in resolution MS^E^-mode with both ESI+ and ESI− acquisition. Chromatography was performed in HILIC mode by gradient elution using a BEH Amide stationary phase (50 × 2.1 mm, 1.7-μm particle size, Waters, Milford, MA, USA). Mobile phase composition was acetonitrile and water with 5 mM ammonium formate and 0.065% formic acid. Data quality was assessed using data acquired from repeated injections at intervals of the QC sample (*n* = 15, corresponding to ~16% of injections) according to previously described methods (Sangster et al., 2006; Engskog et al., 2016). Data preprocessing was performed in the R statistical language (version 3.3.2) using the XCMS package (Smith et al., 2006) followed by normalization to median fold change and filtering of features by retention time and variance in repeated injections. Multivariate data analysis was performed in SIMCA software (ver. 14.1, Umetrics, Umeå, Sweden). Identification of metabolites was achieved by comparing retention time and mass spectral data against in-house and public databases such as the METLIN database (Smith et al., 2005) and Human Metabolome Database (HMDb) (Wishart et al., 2007, 2009, 2013).

### Morphological analysis

After perilymph sampling in the Cispt and Cispt+H_2_ groups or after the last ABR measurement in the H_2_ and Control groups, the animal was deeply anesthetized with sodium pentobarbital (25 mg/kg, intraperitoneally) and subsequently decapitated. The temporal bones were removed, and the bullae were opened to expose the cochleae. Small fenestrations were made in the apex and round window (RW), and 4% phosphate-buffered formaldehyde was gently flushed through the cochlea. The left and right ears were used for surface preparation and cryosectioning, respectively.

Surface preparation was performed as previously described (Canlon and Fransson, 1995). Briefly, the bone surrounding the organ of Corti, stria vascularis, spiral ligament, and tectorial membrane was removed. The tissue was rinsed in phosphate-buffered saline (PBS) several times before it was placed in a solution of 1% bovine serum albumin (BSA) and 0.3% Triton-X100 for 10 min. It was then rinsed, incubated with fluorescent-labeled phalloidin (TRITC 1:200, Sigma-Aldrich) for 45 min, and subsequently rinsed multiple times. The organ of Corti was dissected in approximately 3-mm-long sections, which were placed on microscope slides in glycerol, covered with a coverslip, and sealed with nail polish. All IHCs and OHCs throughout the cochlea were examined using a Zeiss Axio Observer.Z1 microscope (Carl Zeiss, Germany) equipped with a ×40 objective. A reticule placed in the focus of the microscope eyepiece allowed for 0.25 mm of the coil to be viewed and analyzed at one time. After analyzing all hair cells and scar formations, the percentage of hair cell loss per millimeter was calculated and plotted on a cochleogram.

The right ear was decalcified in 0.1 M EDTA, rinsed, and placed in 15% sucrose for 1 day followed by a gradual infiltration of 15% sucrose and OCT Cryomount (Histolab, Sweden) for 4 days, ending with only OCT overnight before embedding in OCT and sectioning on a cryostat in 12-μm-thick sections throughout the cochlea. The sections were mounted on SuperFrost Plus slides (Menzel-Gläser, Germany).

### Immunohistochemistry

To investigate levels of synaptophysin, OCT2, and CTR1, immunohistochemistry labeling was performed in samples from the Cispt, Cispt+H_2_, and Control groups. The antibodies used were monoclonal anti-synaptophysin (mouse igG1 isotype, diluted 1:100; Sigma-Aldrich Inc., USA), rabbit anti-rat organic cation transporter OCT-2 (diluted 1:100; Alpha Diagnostic International, USA), and polyclonal rabbit SLC31A1/CTR1 antibody (diluted 1:500; Novus Biologicals, UK).

All staining procedures except rinsing were carried out in a humidified chamber. The slides were incubated at room temperature for 45 min followed by 15 min in 0.1 M PBS. All slides were treated with 0.3% Triton X-100 for 30 min and then rinsed 3 × 5 min in PBS. Thereafter, blocking was performed in 1% BSA with 5% normal goat serum for synaptophysin and in 1% BSA with 5% normal donkey serum for OCT2 and CTR1. The slides were rinsed and then incubated with the primary antibody overnight at 4°C. The following day, the slides were rinsed 3 × 5 min and then incubated with the secondary antibody, which was Alexa 546 (Life Technologies, USA) for synaptophysin, Alexa 488 (Life Technologies, USA) for OCT2, and Dylight 594 (Jackson ImmunoResearch, USA) for CTR1. All secondary antibodies were diluted 1:400 with blocking solution. After the last rinse (3 × 10 min), a cover slip was mounted with Mowiol (Sigma-Aldrich Inc.) mounting medium.

### Immunohistochemistry analysis

**Figure 1:**
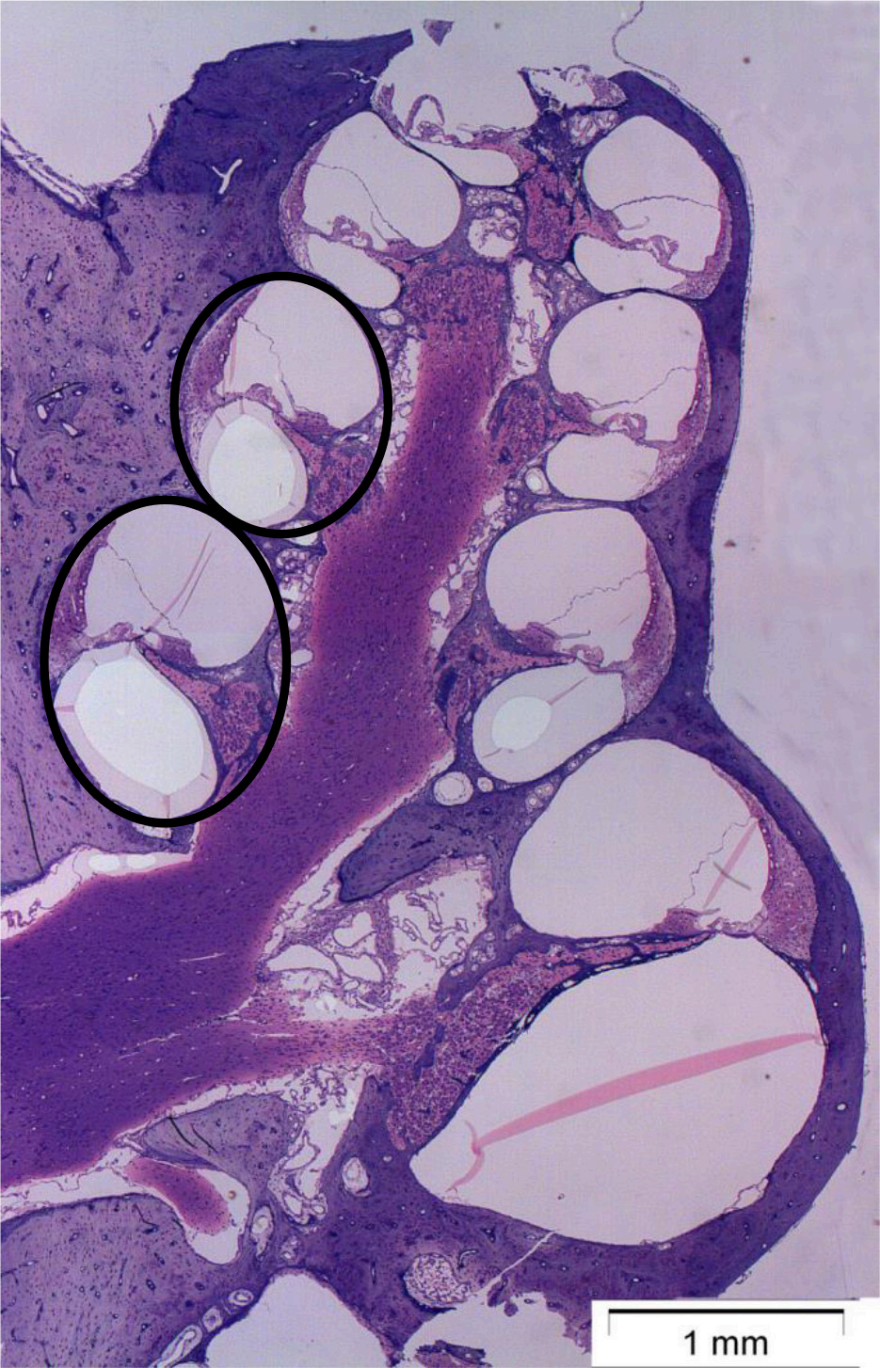
Micrograph showing a mid-modiolar cross-section of a normal guinea pig cochlea. The circles indicate the two points on the basilar membrane, 12 mm (middle turn) and 7 mm (basal turn) from the round window (RW), where the immunoreactivity of synaptophysin, organic cation transporter 2 (OCT2), and copper transporter 1 (CTR1) were analyzed.

Quantification of synaptophysin, OCT2, and CTR1 was performed with densitometry measurement. The cochlea was analyzed at two points on the basilar membrane, 12 mm (middle turn) and 7 mm (basal turn) from the RW (Figure 1), which correlate tonotopically to the frequencies 3.15 and 12.5 kHz (von Békésy, 1960). The images were collected and processed using ImageJ software (1.51j8; National Institutes of Health, Bethesda, MD, USA) (Schneider et al., 2012). All sections were stained at the same time, and all images were taken within 5 days of processing staining.

**Figure 2:**
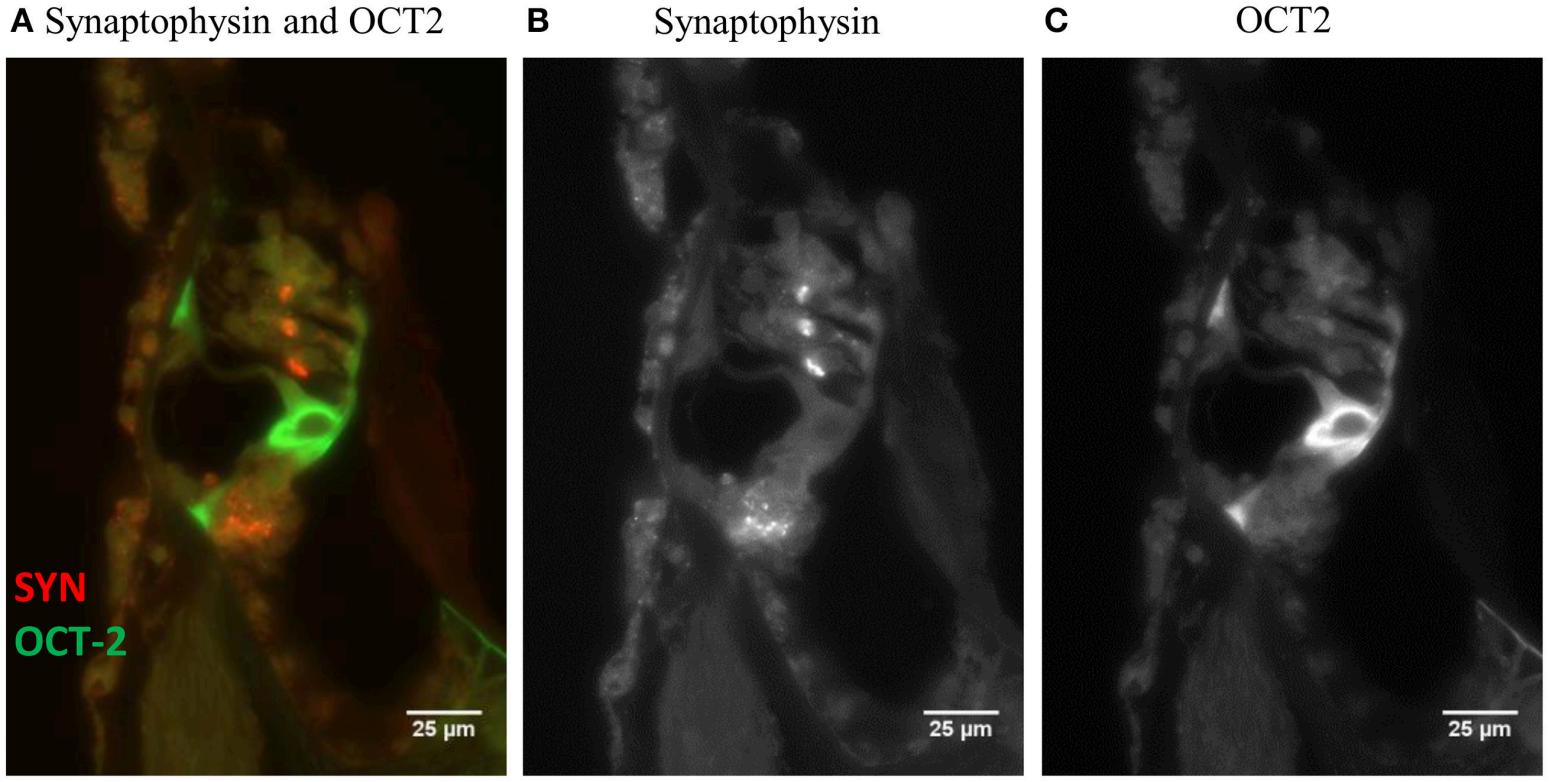
**(A)** A fluorescent image (40 X) of the organ of Corti from a normal guinea pig. The orange and green labeling indicates immunoreactivity of synaptophysin and OCT2. In order to perform a quantification using densitometry measuring, the software converts the colors to a gray scale, **(B)** synaptophysin and **(C)** OCT2, (CTR1 image not shown). Scale bar: 25 μm.

Figure 2A shows a fluorescent image of the organ of Corti from a guinea pig in the Control group stained for synaptophysin and OCT2. Synaptophysin immunoreactivity in the synapses around the IHCs and OHCs are orange, while OCT2 immunoreactivity, which was particularly strong in the inner and outer pillar cells, is green. The software converts the colors to a gray scale (Figures 2B,C) and measures pixel intensity ranging from 0 (black) to 255 (white). These counts were used to quantify the fluorescent intensity of the different regions of interest (ROIs), and the values obtained from the densitometry measurements are referred to as intensity.

The outlines of ROIs were manually traced. The ROI for synaptophysin was the synapse area at the IHCs and OHCs. The ROI for the three rows of OHCs was collected together and calculated as one ROI. The ROI for OCT2 included the inner and outer pillar cells and stria vascularis. The ROI for CTR1 was the synapse area at the IHCs, the Deiters' cells, and the stria vascularis.

For each animal, three sections were analyzed using the densitometry method, and the mean was used to calculate the mean intensity of each group.

### Statistics

For each guinea pig, the frequency-specific electrophysiological hearing threshold obtained before treatment was subtracted from that obtained after 96 h. A mixed-design analysis of variance (ANOVA) was conducted to measure the influence of treatment group and frequency on threshold shift after verifying that the assumptions of fairly symmetrical data from populations with equal variances were met. Two treatment groups were included in the analysis (Cispt and Cispt+H_2_), and frequency consisted of five levels: 3.15, 6.30, 12.5, 20.0, and 30.0 kHz. *Post-hoc* analysis was carried out with the Bonferroni test. Statistical analyses were performed using SPSS (v 22, release 22.0.0.1, IBM Corp., USA). To evaluate hair cell loss and immunohistochemistry data, one-way ANOVA was conducted followed by *post-hoc* analysis with the Holm-Sidak method in Sigma (v 13.0, Systat Inc., USA). A two-sided alpha level of 0.05 was used.

## Results

The median weight change from the day of cisplatin injection to 96 h after cisplatin injection was −9 g (range: −37–9 g) in the Cispt group and −18 g (−49–10 g) in the Cispt+H_2_ group (*p* > 0.05). The values in the H_2_ group and two animals in the Control group used for ABR recording were 14 g (2–26 g) and 29 g (26 and 32 g), respectively. Thus, H_2_ inhalation did not prevent cisplatin-induced weight loss.

### ABR

**Figure 3:**
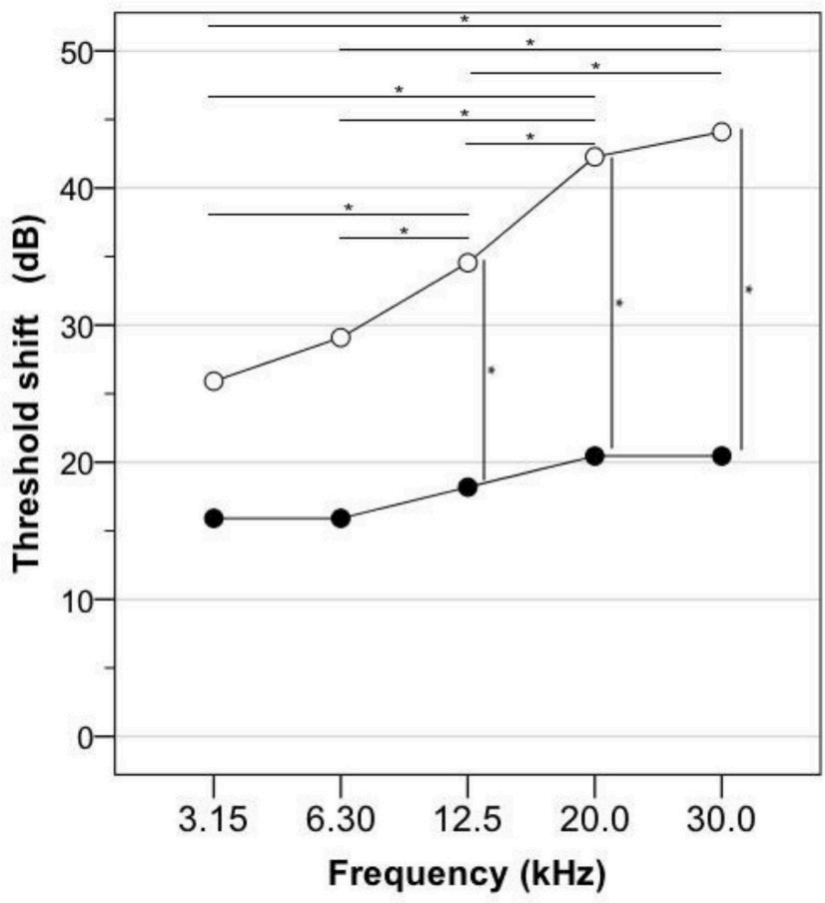
Guinea pigs were subjected to frequency-specific ABR assessment in the left ear before and 96 h after cisplatin injection (8 mg/kg b.w., i.v.). The graph shows the marginal means of the threshold elevations obtained in the Cispt group (*n* = 11; empty circles), which received cisplatin, and in the Cispt+H_2_ group (*n* = 11; filled circles), which received cisplatin immediately followed by inhalation of H_2_ (2% in air) during 60 min. The horizontal lines with an asterisk indicate a significant difference (*p* < 0.05) between two frequencies, which was obtained only in the Cispt group. The vertical lines with an asterisk indicate a frequency-specific significant difference (*p* < 0.05) between the Cispt group and the Cispt+H_2_ group.

The differences between the frequency-specific electrophysiological thresholds obtained before and 96 h after cisplatin injection in the Cispt group and the Cispt+H_2_ is presented in Table 1 and the marginal means of threshold shifts are illustrated in Figure 3. There was a significant difference across the five frequencies [*F*_(2.01, 40.12)_ = 19.34, *p* > 0.001] and between the Cispt and Cispt+H_2_ groups [*F*_(1, 20)_ = 6.81, *p* = 0.017] in threshold shift. There was also a significant interaction between frequency and group [*F*_(2.01, 40.12)_ = 6.10, *p* = 0.005]. *Post-hoc* analysis showed that the threshold shift differed significantly (*p* < 0.05) between different frequencies in the Cispt group but not the Cispt+H_2_ group (Figure 3). It also showed that the frequency-specific threshold shift was significantly different (*p* < 0.05) between the two groups at 12.5, 20.0, and 30.0 kHz (Figure 3). No significant threshold shift was found in the H_2_ and Control animals (data not shown).

**Table 1:** Difference in frequency specific hearing thresholds measured before and 96 h after i.v. injection of cisplatin (8 mg/kg b.w.) to guinea pigs treated with cisplatin only (Cispt group; *n* = 11) or co-treated with hydrogen gas (Cispt+H_2_ group; *n* = 11).

	**Threshold shift (dB)**			
	**Cispt**		**Cispt+H_2_**	
**Frequency (kHz)**	**Mean**	***SD***	**Mean**	***SD***
3.15	25.9	16.3	15.9	13.2
6.30	29.1	16.3	15.9	16.7
12.5	34.5	14.2	18.2	17.6
20.0	42.3	15.4	20.5	19.0
30.0	44.1	12.8	20.5	17.7

### Metabolomics in scala tympani perilymph

**Figure 4:**
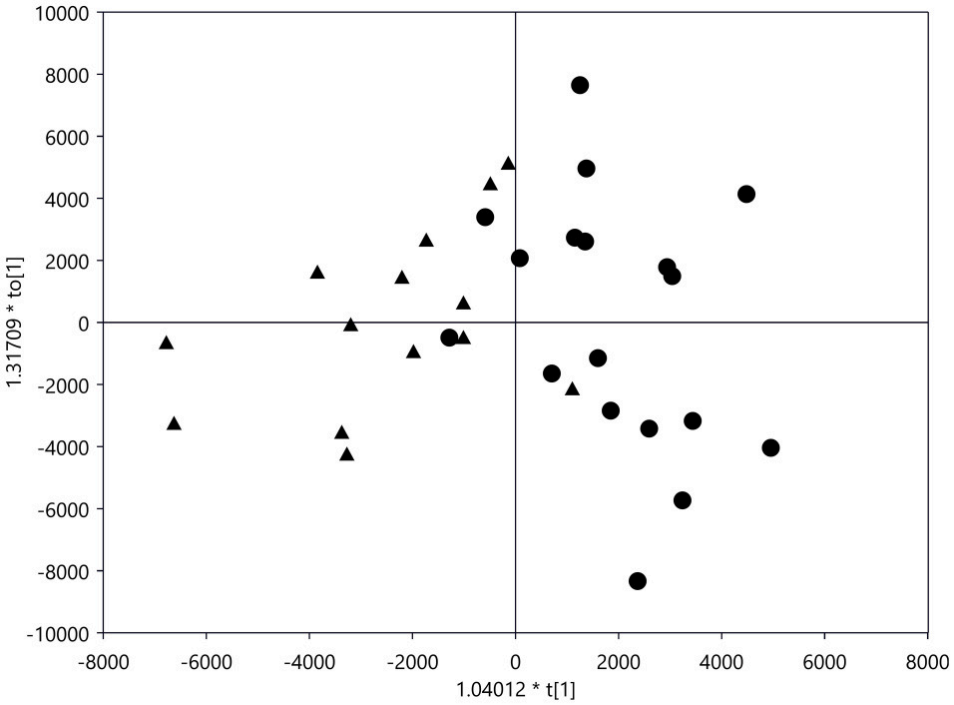
OPLS-DA scores plot (R2X 0.219, R2Y 0.375, Q2 0.222 in the predictive component) of sample Cispt group (triangles) and sample Cispt+H_2_ group (circles).

A metabolomics analysis was performed on 14 samples from 10 animals in the Cispt group and 18 samples from 11 animals in the Cispt+H_2_ group. Following feature filtering, ~1100 features were used in the multivariate models. Orthogonal Partial Least Squares Discriminant Analysis (OPLS-DA) (Trygg and Wold, 2002) was used to model the difference between the sample groups (R2X 0.219, R2Y 0.375, Q2 0.222 in the predictive component). Figure 4 shows the sample group separation along the predictive component in the corresponding OPLS-DA scores plot. A total of 50 metabolites were selected by the models as important for class separation using the S-plot for variable selection (Wiklund et al., 2008). Of these, 30 could be putatively identified using database searches. These include choline sulfate, creatinine, methylguanine, methylguanosine, numerous carnitines, proline betaine, methylcytosine, ecgonine, hydroxystachydrine, homostachydrine, creatine, and dimethylarginine.

### Morphological analysis

#### Quantification of hair cell loss

**Figure 5:**
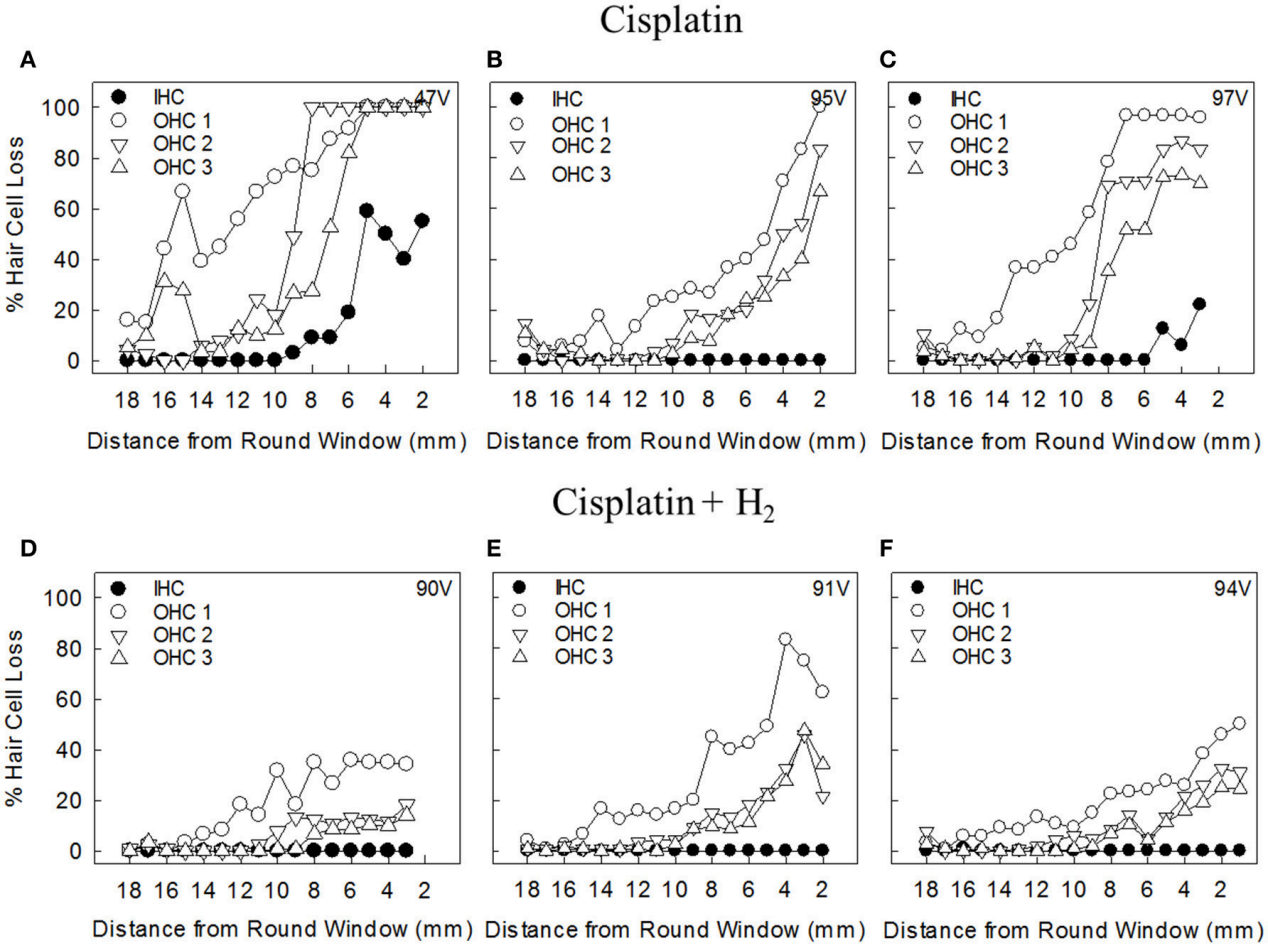
Representative cochleograms from animals in **(A–C)** the Cispt group and **(D–F)** the Cispt+H_2_ group. IHC, inner hair cells; OHC 1, outer hair cells of the first row; OHC 2, outer hair cells of the second row; OHC 3, outer hair cells of the third row.

The morphological changes induced by a single i.v. cisplatin injection were most notable in the basal turn of the cochlea, demonstrating good concordance with the ABR measurements. In the Cispt group, all animals except one showed major OHC loss that was most prominent in the first row. OHC loss started approximately 12 mm from the RW and increased closer to the RW. In this group, five animals showed varying degrees of IHC loss in the basal part of the cochlea. Figures 5A–C shows representative cochleograms from animals in the Cispt group.

OHC loss in the Cispt+H_2_ group was less pronounced, and no IHC loss was observed. With the exception of one animal, OHC loss was considerably decreased throughout the basilar membrane. The first row of OHC was most affected in this group. In the second and third rows, hair cell loss did not rise above 30% in most animals. The hair cell loss started at 10–12 mm from the RW. Figures 5D–F shows representative cochleograms from the Cispt+H_2_ group.

**Figure 6:**
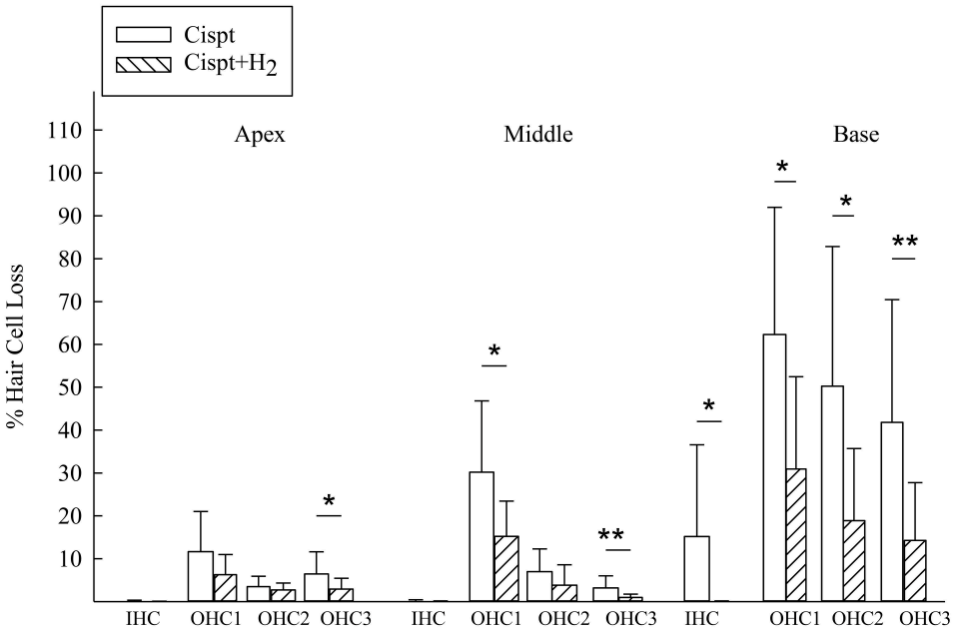
The percentage of lost IHCs and OHCs in the Cispt and Cispt+H_2_ groups was summarized in three different regions, Apex (18-14.1 mm from RW), Middle (14-9.1 mm from RW), and Base (9-2.1 mm from RW). ^*^p < 0.05 and ^**^p < 0.01.

Figure 6 shows the percentages of lost IHCs and OHCs in the first, second, and third rows in the apical, middle, and basal parts of the cochlea. Significantly less loss was noted in the Cispt+H_2_ group.

There was a significant difference between the Cispt and Cispt+H_2_ groups in total OHC (*p* < 0.01) and IHC (*p* < 0.05) loss, with more intact cells in the Cispt+H_2_ group.

#### Immunoreactivity in the control group

**Figure 7:**
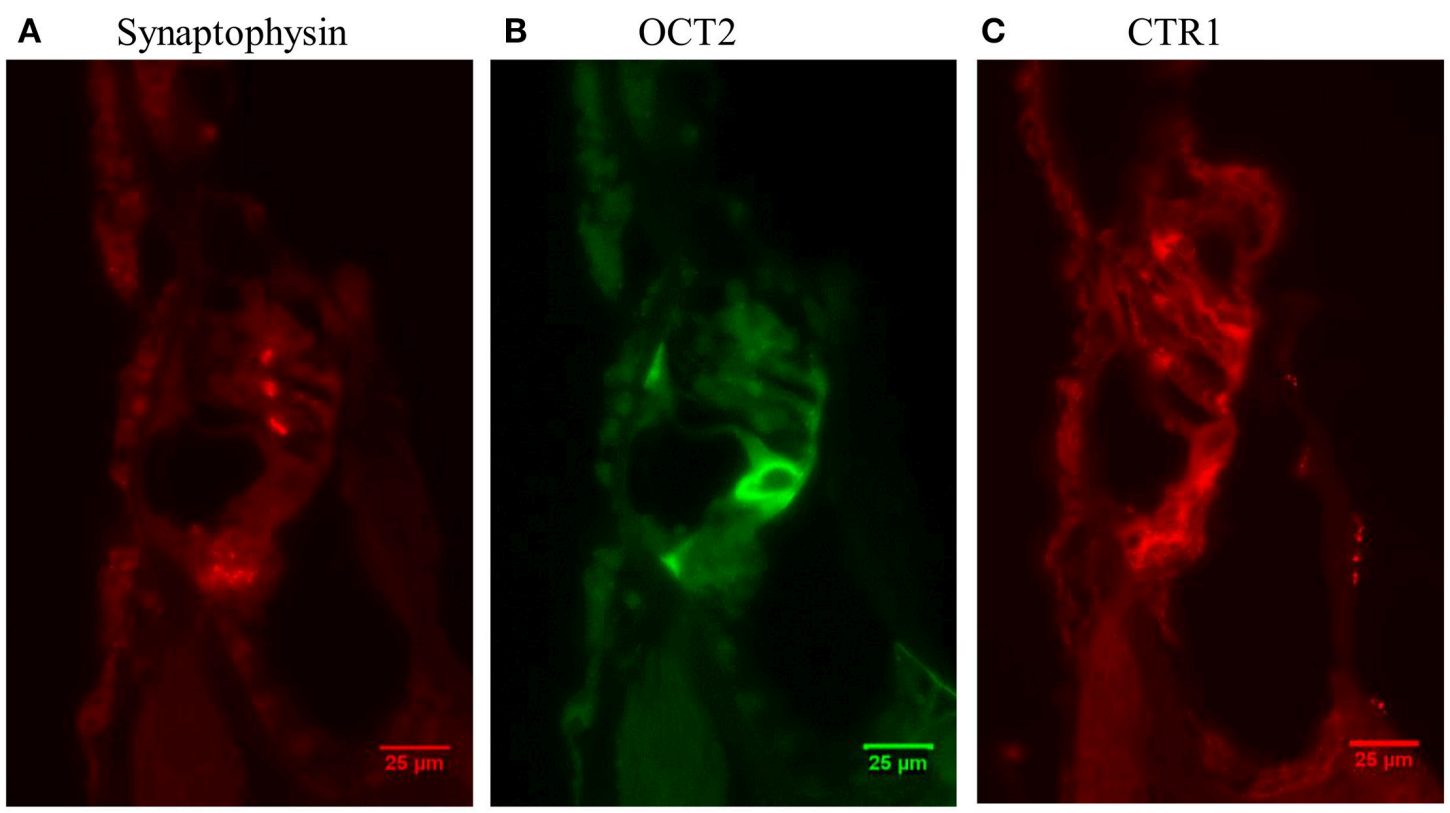
Micrograph (40 X) from a normal guinea pig cochlea (Control group) showing immunoreactivity of **(A)** synaptophysin, **(B)** OCT2, and **(C)** CTR1.

Three different antibodies were used in this study: synaptophysin, OCT2, and, CTR1. In the Control group, synaptophysin immunoreactivity appeared relatively small and distinct areas under each OHC row (Figure 7A). The area of synaptophysin under the IHCs was much larger and lacked distinct borders (Figure 7A). OCT2 signal was clearly seen in the inner and outer pillar cells (Figure 7B) and was also found in the stria vascularis. CTR1 immunoreactivity was observed in the IHC synapse area, Deiters' cells (Figure 7C), and stria vascularis.

#### Synaptophysin

**Figure 8:**
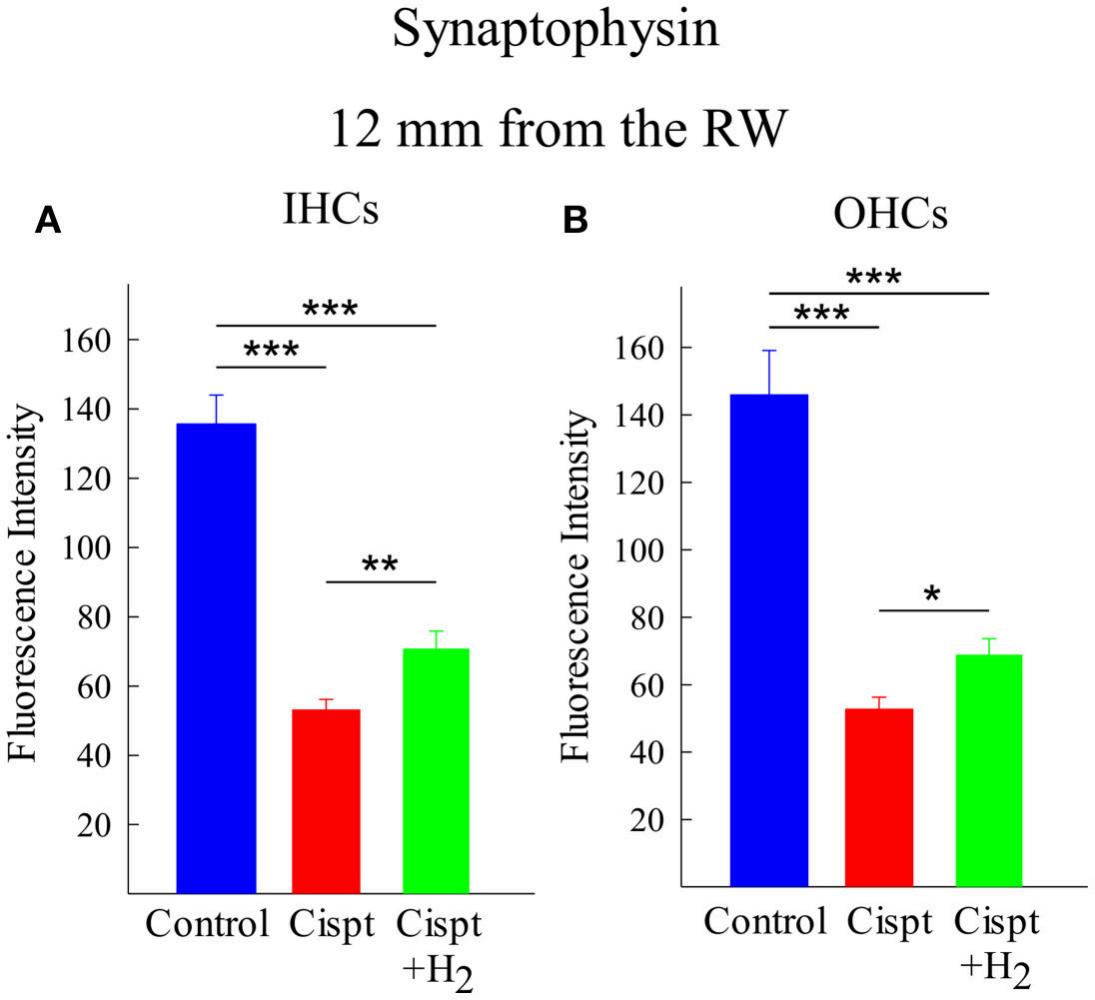
Quantification of the immunoreactivity of synaptophysin, using densitometry, in the synapse area of **(A)** IHCs and **(B)** OHCs at a distance of 12 mm from the RW in the Control group (blue), Cispt group (red), and Cisp+H_2_ group (green). The bars illustrate the mean intensity and the error bars represent SD. ^*^p < 0.05, ^**^p < 0.01, and ^***^p < 0.001.

In the region 12 mm from the RW, there was moderate hair cell loss in the Cispt group and almost no loss in the Cispt+H_2_ group. There was a significant difference between these groups in the synapse area of the IHCs (*p* < 0.01, Figure 8A) and OHCs (*p* < 0.05, Figure 8B). There was also a significant (*p* < 0.001) reduction in the immunoreactivities of both groups compared to Control animals (Figures 8A,B).

In the region 7 mm from the RW, there was major hair cell loss in the Cispt group, and the organ of Corti had collapsed in some of these animals. Synaptohysin immunoreactivity was seen in most the animals; however, there were too few remaining synapses in the OHC area to perform any statistical comparison with the Cispt+H_2_ group. Importantly, immunoreactivity in the Cispt+H_2_ was much higher, and synapses were visible around most OHCs. Although the synapse area was reduced, it was still very distinct, but there was a reduction in immunoreactivity compared to the Control group. No statistical difference was found in the IHC synapse areas between the Cispt and Cispt+H_2_ groups.

### OCT2

**Figure 9:**
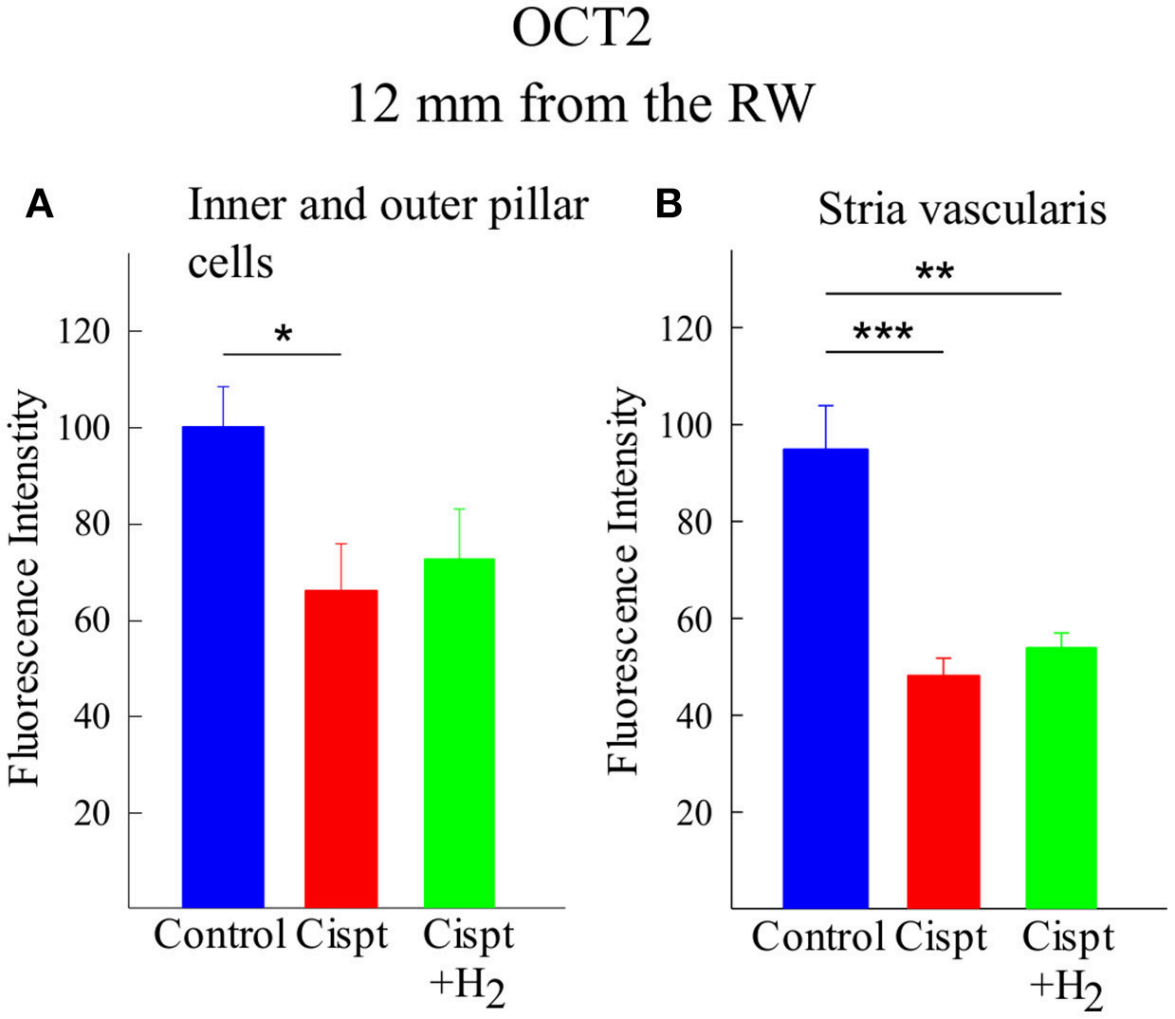
Quantification of the immunoreactivity of OCT2, using densitometry, in **(A)** inner and outer pillar cells and **(B)** stria vascularis at a distance of 12 mm from the RW in the Control (blue), Cispt (red), and Cispt+H_2_ (green) groups. The bars illustrate the mean intensity obtained in the three groups and the error bars represent SD. ^*^p < 0.05, ^**^p < 0.01, and ^***^p < 0.001.

In the inner and outer pillar cells 12 mm from the RW, there was a significant difference (*p* < 0.05) between the Control and Cispt groups in OCT2 immunoreactivity, but no difference was found between the Cispt and Cispt+H_2_ groups (Figure 9A). In the stria vascularis, there was a significant difference between the Control and Cispt groups (*p* < 0.001, Figure 9B) and the Control and the Cispt+H_2_ groups (*p* < 0.01, Figure 9B). In the 7-mm region from the RW, there was a significant decrease (*p* < 0.05) in immunoreactivity of the inner and outer pillar cells in the Cispt group compared to the Control group. In the stria vascularis, there was a significant decrease in both the Cispt and Cispt+H_2_ groups compared to Controls (both *p* < 0.001).

### CTR1

**Figure 10:**
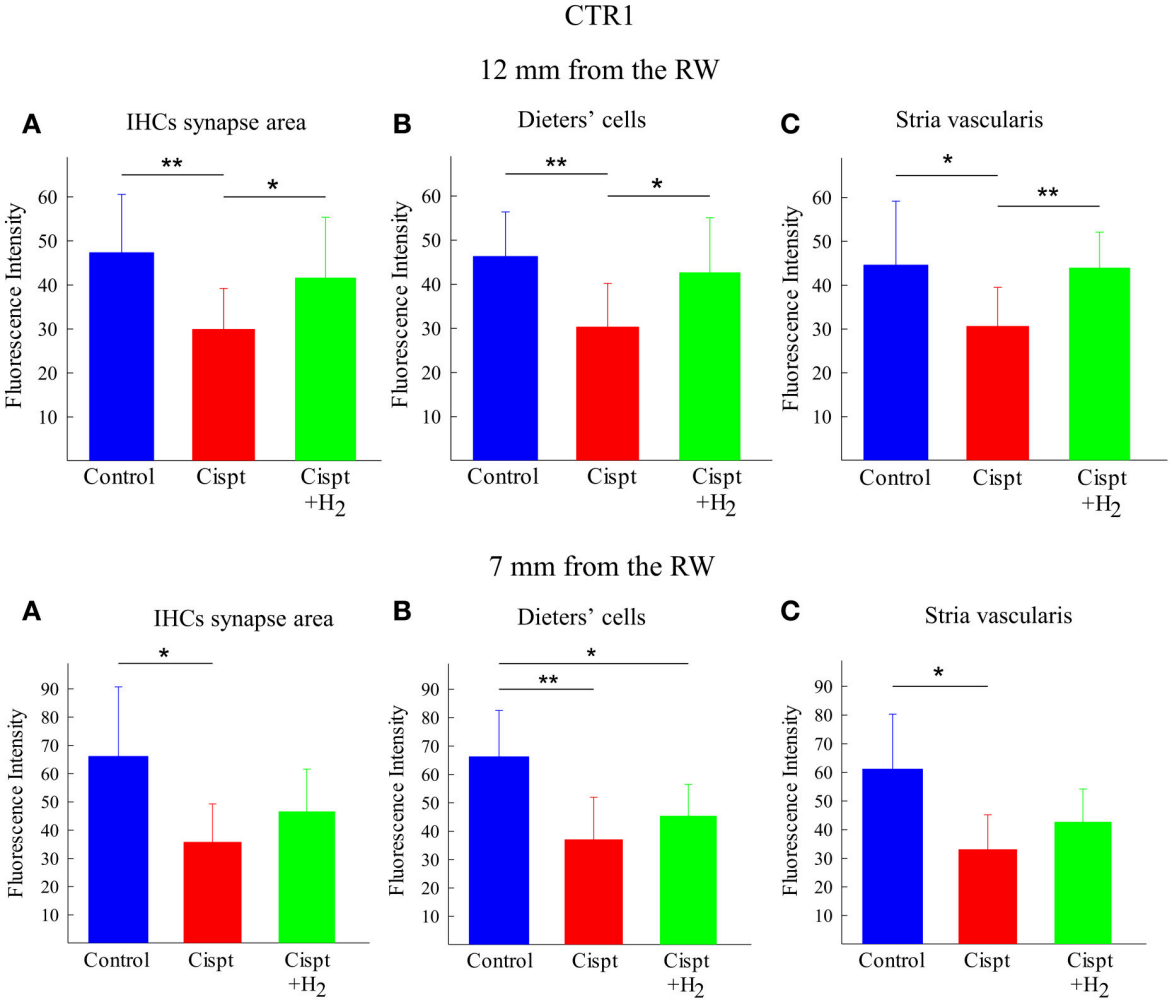
Quantification of immunoreactivity of CTR1, using densitometry. Upper row show the results from the 12 mm region from the RW and the lower row show the results from the 7 mm region from the RW. **(A)** The synapse area of IHCs, **(B)** Dieters' cells, and **(C)** stria vascularis of the cochlea in the control (blue), Cispt (red), and Cispt+H_2_ (green) groups. The bars illustrate the mean intensity obtained in the three groups and the error bars represent SD. ^*^p < 0.05 and ^**^p < 0.01.

CTR1 immunoreactivity in the synapse area of IHCs, the Deiters' cells, and the stria vascularis was calculated at 12 and 7 mm from the RW. In the 12-mm region, there was higher immunoreactivity in all areas observed in the Cispt+H_2_ group compared to the Cispt group. No significant difference was found in the 7-mm region between the two groups (Figure 10).

## Discussion

High-dose cisplatin treatment is associated with several toxic insults to the cochlea. Antioxidants were previously shown to protect cochlear structures under experimental conditions. Both systemic and local administration have been employed and demonstrated benefits in experimental animals. Here, we investigated the otoprotective potential of gaseous H_2_ given for 60 min starting immediately after albino guinea pigs received a single cisplatin injection. Cisplatin administration caused electrophysiological hearing threshold shifts that were largest at high frequencies. The shifts were partly prevented by H_2_ inhalation. The hair cell counts corresponded with the hearing threshold shifts and showed substantial hair cell loss that declined from the base to apex. As previously found (Berglin et al., 2011), OHC loss was greatest in the first row, less in the second row, and least in the third row. There was also some IHC loss in the basal turn of the cochlea, which is less common in cisplatin-treated animals. H_2_ inhalation had an important protective effect on OHCs and completely prevented IHC loss. These results agree with those of a previous study, where inhalation of H_2_ reduced ABR-assessed threshold shifts and hair cell loss in cisplatin-treated rats (Qu et al., 2012b).

Assessment of threshold shifts and IHC/OHC loss are two rough methods to measure the value of an otoprotective treatment. New, more refined measures are needed to better understand as to the effects of ototoxicologic treatments on cellular and subcellular levels. New insights from animal experiments suggest that synaptopathy plays a key role in hearing damage due to noise, aging, and aminoglycoside treatment, and that it can occur even when hearing thresholds still are normal and hair cells are preserved (Liberman and Kujawa, 2017). These observations indicate that synaptopathy could be an endpoint measure to assess ototoxicological risk and protective treatments. We explored whether synaptopathy may be involved in cisplatin-induced ototoxicity and if it could be prevented by gaseous H_2_. We assessed the effect of cisplatin administration without and with concomitant H_2_ inhalation on synaptophysin immunoreactivity at the cochlear nerve terminals around the IHCs and OHCs. Measurements were performed 7 and 12 mm from the RW, approximately correlating tonotopically to 12.5 and 3.15 kHz frequencies, respectively (von Békésy, 1960). Although most synapses link IHCs and type I afferent neurons, synapses at the OHCs were also assessed as the OHCs may undergo apoptosis after cisplatin administration. Densitometry analysis was performed to provide a semi-quantitative measure of synaptophysin staining. In many cases, the large destructive effects of cisplatin treatment resulted in collapse of the organ of Corti in the basal turn of the cochlea, which severely hampered the immunohistochemical evaluation at 7 mm. The ROI of synaptophysin was the synapse area of the hair cells. At 12 mm, where threshold changes and OHC loss were less noticeable than at 7 mm and where there was no IHC loss, cisplatin administration still reduced synaptophysin labeling. Notably, this was less pronounced in H_2_-treated animals. Evaluation at 7 mm was only possible in the Cispt+H_2_ group; there were too few evaluable samples in the Cispt group due to cisplatin cochleotoxicity. Thus, our study shows that synaptopathy might play a role in cisplatin-induced hearing damage. Reduced synaptophysin levels in the inner ear were previously associated with amikacin-induced ototoxicity (Ernfors et al., 1996) and noise trauma (Canlon et al., 1999) in guinea pigs and with aging in mice (Bartolome et al., 2009). Although H_2_ inhalation ameliorated the cisplatin-induced change in synaptophysin staining, its protective effect using a post-treatment mode was incomplete. A possible explanation for why partial protection was observed is suboptimal timing. Cisplatin was detected in the scala tympani perilymph just 10 min after i.v. administration in guinea pigs (Laurell et al., 1995; Hellberg et al., 2013). Considering cisplatin's high reactivity, it is possible that a protective agent must already be present in the cochlea to prevent ototoxicity. A second possible explanation is suboptimal dosing. Recognizing the limitations of the methodology and difficulties of knowing the exact pharmacokinetics and pharmacodynamics of H_2_ and cisplatin in the inner ear, the results still indicate that H_2_ is a promising otoprotective candidate.

Cisplatin is rapidly distributed to the cochlea after systemic administration (Laurell et al., 1995; Hellberg et al., 2013). The details of this transport remain to be elucidated. A study in mice suggested the involvement of the transport protein OCT2 for cochlear uptake of cisplatin (Ciarimboli et al., 2010). Further support for a role of OCT2 was provided by two recent clinical studies that showed an association between a specific gene variant of the OCT2 gene *SLC22A2* and reduced ototoxicity in cisplatin-treated patients (Lanvers-Kaminsky et al., 2015; Spracklen et al., 2016). We therefore wanted to explore the effect of cisplatin administration on OCT2 in the cochlea and whether it was altered by concomitant H_2_ inhalation. The ROI of OCT2 included the supporting cells of the organ of Corti (mainly the inner and outer pillar cells) and the stria vascularis. Cisplatin administration reduced OCT2 immunolabeling at 12 mm in both regions, effects that were not prevented by H_2_ inhalation. Evaluation at 7 mm was only possible in the Cispt+H_2_ group due to extensive cochleotoxicity in the Cispt group. We can only speculate about the causes of reduced OCT2 staining. Possible explanations are that cisplatin alters protein synthesis of OCT2 or promotes the apoptosis of OCT2-expressing cells. Another possibility is that cisplatin binds to OCT2 and thereby impairs binding of the OCT2 antibody. The consequences of reduced OCT2 immunolabeling in the cochlea also warrants further investigation. The influence of OCT2 expression on cisplatin-induced nephrotoxicity was first suggested about a decade ago (Yonezawa et al., 2005) and, as recently reviewed, there are many shared feature between cisplatin's ototoxic and nephrotoxic effects (Karasawa and Steyger, 2015). OCT2 is also a determinant of the efficacy of cisplatin-based cancer treatment (Liu et al., 2012). With regard to OCT2 cochlear localization, others have shown that OCT2 can be found in the mouse stria vascularis (Ciarimboli et al., 2010; More et al., 2010) and in IHCs and OHCs (Ciarimboli et al., 2010). Our previous study of guinea pigs revealed OCT2 in the supporting cells of the organ of Corti when using paraffin-sectioning (Hellberg et al., 2015) in contrast to cryosectioning, which was used in the present investigation and in mouse studies (Ciarimboli et al., 2010; More et al., 2010). The tunnel of Corti bordered by the inner and outer pillar cells communicates with the perilymphatic space, and OCT2 expression in these cells indicates that active uptake of cisplatin through this pathway to the endolymphatic space may contribute to OHC toxicity.

Another transporter implicated in cisplatin-induced ototoxicity is CTR1. This protein is abundantly expressed in the mouse cochlea, and pretreatment with the CTR1 substrate copper sulfate decreased cisplatin-induced ototoxicity (More et al., 2010). The ROI of CTR1 was the synapse area of the IHC, Deiters' cells, and the stria vascularis. Cisplatin treatment reduced CTR1 immunolabeling at 12 mm in all these regions, an effect that was prevented by H_2_ inhalation. The reduction pattern was similar in the 7-mm region, but the difference between the Cispt and Cispt+H_2_ groups did not reach statistical significance. As for the reduction of OCT2 immunolabeling, we can only hypothesize about the causes and consequences of our CTR1 findings. Clinical support for a role of CTR1 in cisplatin-induced ototoxicity remains sparse (Xu et al., 2012), but its importance for cisplatin-induced anticancer efficacy is more established (Liu et al., 2012). There are a number of copper influx and efflux transporters, and several have been shown to be involved in the cellular passage of cisplatin. Future studies that simultaneously investigate both types of copper transporters might increase our understanding in this field. With regard to CTR1 localization within the cochlea, CTR1 was abundantly expressed in the stria vascularis, OHCs, IHCs, and supporting cells in a previous mouse study (More et al., 2010).

As the precise trafficking of cisplatin within the cochlea is not clear, we wanted to further explore the effect of gaseous H_2_ on scala tympani perilymph. We aspirated scala tympani perilymph for metabolomics analysis. Although preliminary, the data in the OPLS-DA score plot indicate a clear separation of the Cispt and Cispt+H_2_ groups. This finding further supports the possibility that H_2_ inhalation can reduce the ototoxic effect of cisplatin within the cochlea before the drug affects the OHCs (i.e., a preventive mechanism before the drug reaches the organ of Corti). This is most likely in line with strategies to prevent cisplatin ototoxicity by intratympanic antioxidant administration. We recently showed that injection of an ototoxic dose of cisplatin to guinea pigs induces a wide range of changes in the blood metabolome (Videhult Pierre et al., 2017).

It is reasonable to speculate that there are important differences between ototoxicity and otoprotection in healthy experimental animals and human beings treated for platinum-sensitive cancer. A major limitation of the study is the focus on the cochlear effects of H_2_ administration without an oncologic perspective. We did not evaluate any influence of H_2_ on cisplatin cytotoxicity in a tumor target. In cancer research, it is important to assess antitumor activity when introducing a novel co-administrated compound to reduce side effects. Moreover, the hearing situation of patients undergoing cisplatin treatment is more complex, with a number of confounding factors influencing the ototoxic effect. Caution must be taken when interpreting our results obtained in guinea pigs. However, the *in vivo* model reflects a clinically relevant question, although cisplatin administration routes and methods differ from that in clinical practice. For example, we administered a bolus injection of cisplatin, whereas repeated injections are usually given in clinical practice.

The findings in the present investigation raise a number of interesting questions that have to be further studied. Even though our method could be a potentially attractive approach in clinical settings, several issues must be addressed before it can be used as a proof of concept. Antioxidant treatment may have negative effects on cisplatin cytotoxicity in tumors (Freyer et al., 2017) and could even promote tumor growth (Sayin et al., 2014) and metastasis (Le Gal et al., 2015), so more research is needed to develop strategies that circumvent ototoxicity in patients undergoing cisplatin treatment. Substantial effort will be required to assess the effects of H_2_ with and without cisplatin on tumor growth and spread. One finding that has not received enough attention is cisplatin's effect on IHC synapses. The observed synaptopathy after a single cisplatin injection could be a target for additional research. In the present study, only H_2_ administered after cisplatin was used to prevent ototoxicity. Pre-loading H_2_ could be a more effective way to protect hearing. Apart from the discrepancy between the influence of H_2_ on OCT2 and CTR1 immunoreactivities, one striking finding in this study was that cisplatin altered immunolabeling of the transport proteins in cochlear tissue. Additional work is needed to clarify the active transport processes involved in cisplatin ototoxicity. Nevertheless, our findings suggest that H_2_ administration could prevent cisplatin ototoxicity, but further evaluation is necessary.

## Conclusion

Although cisplatin is an old drug, it is still frequently used in oncology despite its side effects. There have been no clinical breakthroughs for otoprotection during cisplatin therapy. The present results demonstrate that gaseous H_2_ is a promising agent that can protect against cochlear injury. First, H_2_ showed an effect on frequency-specific electrophysiological hearing thresholds. Second, surface preparations of the cochlea revealed that OHC loss was attenuated in H_2_-treated animals. Third, at the subcellular level, H_2_ produced a protective effect on IHC synapses. Hearing loss after cisplatin treatment is multifactorial, and our results implicate synaptopathy as an additional mechanism. Using a guinea pig model for an otoprotective study has some obvious limitations as they cannot be compared with cancer patients. The advantage is the ability to study the inner ear structures in detail after cisplatin treatment, which is not possible in human subjects.

## Author contributions

All authors listed, have made substantial, direct and intellectual contribution to the work, and approved it for publication.

### Conflict of interest statement

The authors declare that the research was conducted in the absence of any commercial or financial relationships that could be construed as a potential conflict of interest.
